# Affine Iterative Closest Point Algorithm Based on Color Information and Correntropy for Precise Point Set Registration

**DOI:** 10.3390/s23146475

**Published:** 2023-07-17

**Authors:** Lexian Liang, Hailong Pei

**Affiliations:** Key Laboratory of Autonomous Systems and Networked Control, Ministry of Education, Unmanned Aerial Vehicle Systems Engineering Technology Research Center of Guangdong, South China University of Technology, Guangzhou 510640, China; 01298469@cimc.com

**Keywords:** point set registration, RGB-D data, iterative closest point, color Information, correntropy

## Abstract

In this paper, we propose a novel affine iterative closest point algorithm based on color information and correntropy, which can effectively deal with the registration problems with a large number of noise and outliers and small deformations in RGB-D datasets. Firstly, to alleviate the problem of low registration accuracy for data with weak geometric structures, we consider introducing color features into traditional affine algorithms to establish more accurate and reliable correspondences. Secondly, we introduce the correntropy measurement to overcome the influence of a large amount of noise and outliers in the RGB-D datasets, thereby further improving the registration accuracy. Experimental results demonstrate that the proposed registration algorithm has higher registration accuracy, with error reduction of almost 10 times, and achieves more stable robustness than other advanced algorithms.

## 1. Introduction

With the rapid development of 3D reconstruction in various fields, such as image processing [1,2,3,4,5,6], machine vision [7,8,9,10,11], and simultaneous localization and mapping (SLAM) [12,13,14], point set registration becomes more and more important, which is a key technique in image registration. The purpose is to match point sets from two or more images obtained at different times, different environments, or in different sensors. After geometric transformations, such as rotation and translation, the transformed point set is consistent in spatial expression. However, in practical applications, there are still several problems in point set registration, including (1) the correspondence between the two sets of points is unknown; and (2) there is insufficient information for point set matching when the local structures are unsalient. Besl and McKay [15] proposed an iterative closest point (ICP) algorithm, which is an efficient algorithm for solving the problem of rigid registration with two point sets.

In recent years, with the widespread application of the ICP algorithm, many scholars have carried out a lot of research around the ICP algorithm to improve it from the aspects of speed, convergence, and robustness. Among them, Blais et al. [16] adopted the method of random sampling of the original point set to boost the registration speed, but could not guarantee the accuracy of registration. Fitzgibbon [17] used the Levenberg–Marquardt algorithm to make the ICP registration algorithm converge faster. Sharp et al. [18] studied the ICP registration algorithm based on invariant features to decrease the probability of the algorithm falling into a local minimum and improve the robustness of the algorithm. However, the algorithm cannot solve the problem of a lot of noise interference in the dataset. For the registration problem of datasets with noise and outliers, Chetverikov et al. [19] proposed a TriICP algorithm to suppress noise and outliers. Phillip et al. [20] improved the trimmed ICP algorithm, which further improved the accuracy of registration. Myronenko et al. [21] proposed the Coherent Point Drift (CPD) algorithm, which used a probabilistic method to register the point sets. The model point set was represented by a Gaussian Mixed Model (GMM), and the target point set was regarded as the observation of the model point set. When these two point sets are registered, the correspondence is the maximum GMM posterior probability, which overcomes noise and outliers. Yang et al. [22] proposed a branch and bound solution based on searching the entire space SE (3), which solved the problem that the ICP algorithm was easily trapped in local extreme values. Although the accuracy of registration is improved, its calculation speed becomes slower. Du et al. [23] established a probabilistic model of the ICP algorithm and proposed the PICP algorithm to improve the robustness of the ICP algorithm.

In addition, some learning-based approaches have been proposed to address partial overlap registration [24,25,26,27]. For example, Zeng et al. [28] proposed a fast rigid registration method based on deep learning for 3D reconstruction, which can also be extended to other registration. RPNet [29] is less sensitive to initialization and more robust for point cloud registration. The network of this method obtains the corresponding soft allocation of points to solve the local overlap of point clouds problem. Deep closest point [30] uses dynamic graph neural network for feature extraction, and uses an attention module to generate features to establish correspondence. OMNet [31] estimates the overlapping masks to reject non-overlapping regions for partial-to-partial point cloud registration. Similarly, FINet [32] leverages the dual branches feature interaction and achieves better registration results. Yu et al. [24] presented a rotation invariant transformer to better address low overlap registration with large rotations. Gu et al. [25] proposed a recurrent framework for the 3D motion estimation to avoid the irregular data structure. Hu et al. [26] proposed a novel deep learning framework to generate omnidirectional 3D point clouds of human bodies by registering the front- and back-facing partial scans captured by a single-depth camera. Sun et al. [27] designed an end-to-end 3D graph deep learning framework of point cloud registration, which can simultaneously learn the detector (graph attention expression) and the descriptor (graph deep feature) for point cloud registration in a weakly supervised way, so that the learned detector and descriptor promote each other in the process of model optimization. Qin et al. [33] proposed Geometric Transformer to learn geometric feature for robust superpoint matching, which encodes pair-wise distances and triplet-wise angles, making it robust in low-overlap cases and invariant to rigid transformation.

However, in the actual application scene, the images to be registered often have some deformation. Therefore, the non-rigid registration problem has become another hot issue for many scholars. For affine registration problems, Feldmar et al. [34] tried to find the best global affine transformation by introducing differential information. Linear combinations of neighbor and similar rigid transformations are used to obtain more accurate local affine transformations between point sets. Amberg et al. [35] constrained the objective function by adding rigid regular terms and labeled terms to solve the affine transformation. Du et al. [36] put forward an affine registration algorithm based on Independent Components Analysis (ICA) to achieve accurate point set registration.

With the development of RGB-D imaging technology, many researchers pay more attention to the color information of the collected data [37]. Men et al. [38] added color information to the spatial information, which improved the registration accuracy and accelerated the convergence speed of the algorithm. Korn et al. [39] proposed an ICP algorithm based on Lab color information based on the Generalized-ICP algorithm, which improved the robustness of registration.

Although the above-mentioned algorithms improve the traditional registration algorithm from various aspects, they cannot match RGB-D data with a lot of noise and slight deformation well. In real scenes, the RGB-D data often contain a large number of moving objects, and the moving objects have a slight deformation during data collection, and the RGB-D data often have some noise and outlier interference, which reduces the registration accuracy. To solve these problems, this paper proposes a precise affine registration with color information and correntropy. First, this paper introduces color information into the algorithm as a feature to further improve the more accurate and reliable correspondence. Secondly, to solve the interference of noise and outliers in the dataset, this paper introduces the correntropy as a distance measurement method, which improves the registration accuracy. Experimental results demonstrate that our registration algorithm has higher registration accuracy and more stable robustness than other registration algorithms.

Overall, the contributions of this paper are summarized as:

- We introduce color information into affine point cloud registration, which can increase the robustness of the algorithm.
- we introduce the robust correntropy metric to address outliers and noises in the point clouds for more accurate registration.

The rest of this paper is organized as follows. Among them, in Section 2, a review of the traditional affine ICP algorithm. In Section 3, the precise affine registration with color information and correntropy is proposed. In Section 4, we use simulated data and real data to conduct experiments and analyze the experimental results. At last, the conclusion is summarized in the last section.

## 2. A Review of the Traditional Affine ICP Algorithm

The traditional affine ICP algorithm is a very widely used registration algorithm [40,41,42]. We usually define two point sets $M={\left\{{\vec{m}}_{i}\right\}}_{i=1}^{N_{m}}$ and $D={\left\{{\vec{d}}_{j}\right\}}_{j=1}^{N_{d}}$ in a space $R^{n}$. Then, we can establish the objective function of affine registration between the two sets of points to be matched that we define, and give the objective function as the following least squares (LS) problem[43]:

(1) $$\begin{array}{c} \min_{\underset{j\in \{1,2,\cdots ,N_{d}\}}{A,\vec{t},}}\sum_{i=1}^{N_{m}}{∥\left(A{\vec{m}}_{i}+\vec{t}\right)-{\vec{d}}_{j}∥}_{}^{2}\\ s.t.\;\;\;\;\det(A)\neq 0 \end{array}$$

where *A* is the affine matrix, $\vec{t}$ is the translation vector.

The method of solving the algorithm is divided into two steps. Firstly, for any point ${\vec{m}}_{i}$ in the point set *M*, the affine ICP algorithm finds the closest point in the point set *D* as the corresponding point; secondly, we can calculate the affine matrix *A* and translation vector $\vec{t}$ of the current loop; thirdly, the corresponding point information is updated according to the above steps, and the transformation matrix is continuously calculated until the registration error is minimum or the iteration time exceed the maximum number, then the cycle is terminated.

## 3. Precise Affine Registration with Correntropy and Color Information

In this section, we propose a precise affine registration algorithm with color information and correntropy to achieve accurate registration.

### 3.1. Problem Statement

**Figure 1:**
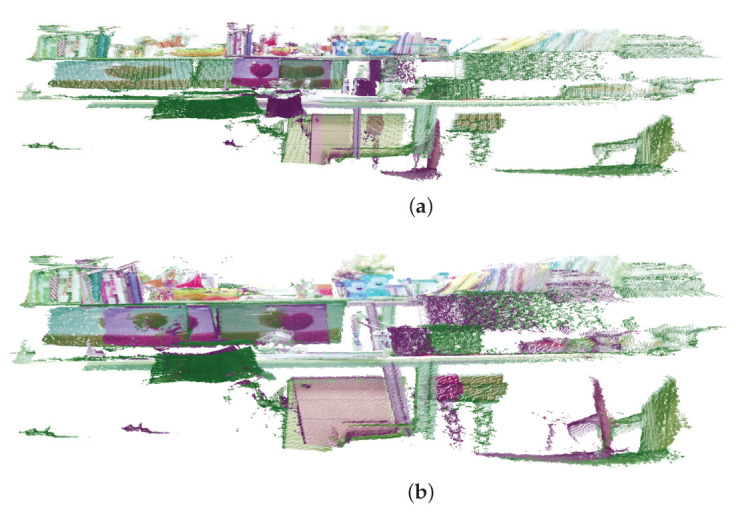
The registration result. (**a**) The original datasets. (**b**) The affine ICP registration result.

In real-world scenarios, collected data from cameras and other equipment may have slight deformations, and some noise data may be mixed in at the same time. Although traditional affine registration can solve the problem of deformation registration of objects, the accuracy of matching weak geometric data with noise and outliers is not high, and Figure 1 verifies this problem. It can be seen from the figure that the affine registration algorithm is not accurate for solving such problems. For weak geometric data, the color information in the image is more obvious, so more reliable correspondences can be found, thereby improving the registration accuracy. Then, to improve the registration accuracy, we can introduce color information as a feature, thereby increasing the registration accuracy for weak geometric data. Therefore, we can get the following formula

(2) $$\begin{array}{c} \min_{A,\vec{t},j\in \left\{1,2,\cdots ,N_{d}\right\}}({∥\left(A{\vec{m}}_{i}+\vec{t}\right)-{\vec{d}}_{j}∥}_{}^{2}\\ +\alpha {\left(r_{i}^{m}-r_{j}^{d}\right)}^{2}+\alpha {\left(g_{i}^{m}-g_{j}^{d}\right)}^{2}+\alpha {\left(b_{i}^{m}-b_{j}^{d}\right)}^{2}) \end{array}$$

where $r_{i}^{m}$, $r_{j}^{d}$, $g_{i}^{m}$, $g_{j}^{d}$, $b_{i}^{m}$, and $b_{j}^{d}$ are the color information of two points, and α represents the weight ratio of color information, which is set to be 0.1. Color information is easily affected by changes in illumination. Therefore, when light changes are more obvious, the parameter α can be adjusted to a smaller value, thus reducing the effect of color errors.

The objective Equation (2) enables a more accurate and reliable correspondence between these two point sets. However, real datasets often contain a lot of noise and outliers, which leads to a reduction in registration accuracy. To solve this problem, this paper introduces a measure of correntropy, which replaces the measure of Euclidean distance measurement in the traditional registration method. The measurement method is as follows

(3) $$\begin{array}{c} s\left({\vec{m}}_{i},{\vec{d}}_{i}\right)=\sum_{i=1}^{N_{m}}\exp\left(-\frac{{∥{\vec{m}}_{i}-{\vec{d}}_{i}∥}_{2}^{2}}{2\sigma^{2}}\right) \end{array}$$

where σ is a free variable that is set to 0.1. It can be known from the above formula that if the distance is larger, the correntropy distance is smaller and the registration result is worse; otherwise, the correntropy distance is larger, and the registration accuracy is higher. In other words, the correntropy metric falls into the robust kernel functions which assign large errors with bigger weights and assign small errors with smaller weights for alleviating the influence of inliers and outliers.

According to the above equation, we can build a new objective function:

(4) $$\begin{array}{c} \max_{A,\vec{t},j\left\{1,2,\cdots ,N_{d}\right\}}\sum_{i=1}^{N_{m}}\exp\left(\begin{array}{c} -({∥\left(A{\vec{m}}_{i}+\vec{t}\right)-{\vec{d}}_{j}∥}_{}^{2}+\alpha {\left(r_{i}^{m}-r_{j}^{d}\right)}^{2}\\ +\alpha {\left(g_{i}^{m}-g_{j}^{d}\right)}^{2}+\alpha {\left(b_{i}^{m}-b_{j}^{d}\right)}^{2})/2\sigma^{2} \end{array}\right)\\ s.t.\;\det\left(A\right)\neq 0 \end{array}$$

### 3.2. Precise Affine Registration with Color Information and Correntropy

To solve the registration problem described by Equation (4), this algorithm uses an iterative solution algorithm similar to the affine ICP algorithm. In each loop, the nearest neighbor search algorithm is used to establish the correspondence between these two point sets, and then the affine transformation is solved according to the correspondence. This algorithm is mainly divided into two steps, which are expressed as follows:

(1) According to the transformation in step (k−1), the corresponding relationship is established between these two point sets:

(5) $$\begin{array}{c} c_{k}\left(i\right)=\underset{j\in \left\{1,2,\cdots ,N_{d}\right\}}{arg\min}({∥\left(A{\vec{m}}_{i}+\vec{t}\right)-{\vec{d}}_{j}∥}_{}^{2}\\ +\alpha {\left(r_{i}^{m}-r_{j}^{d}\right)}^{2}+\alpha {\left(g_{i}^{m}-g_{j}^{d}\right)}^{2}+\alpha {\left(b_{i}^{m}-b_{j}^{d}\right)}^{2}) \end{array}$$

(2) Calculating $\left(A_{k},{\vec{t}}_{k}\right)$ by the correntropy method for step *k*:

(6) $$\begin{array}{c} \left(A_{k},{\vec{t}}_{k}\right)=\underset{A,\vec{t}}{\arg\max}\sum_{i=1}^{N_{m}}\exp(-({∥A{\vec{m}}_{i}+\vec{t}-{\vec{d}}_{c_{k}\left(i\right)}∥}_{2}^{2}+\alpha {\left(r_{i}^{m}-r_{c_{k}\left(i\right)}^{d}\right)}^{2}\\ +\alpha {\left(g_{i}^{m}-g_{c_{k}\left(i\right)}^{d}\right)}^{2}+\alpha {\left(b_{i}^{m}-b_{c_{k}\left(i\right)}^{d}\right)}^{2})/2\sigma^{2}) \end{array}$$

The algorithm iteratively calculates the affine matrix *A* and translation vector $\vec{t}$ to make it approach the optimal solution until the error converges to a preset value or exceeds the maximum number *k* of cycles.

For the problem of correspondence search described in Equation (5), we will use the *k*-d tree-based method [44,45,46] to search the nearest point in three dimensions. For the optimization problem described in Equation (6), we will give detailed algorithm solution steps and closed-form solution of the algorithm.

First, we calculate the approximate solution of $\vec{t}$. Then, Equation (6) is rewritten as follows:

(7) $$\begin{array}{c} F\left(A_{k},{\vec{t}}_{k}\right)=\sum_{i=1}^{N_{m}}\exp(-({∥A{\vec{m}}_{i}+\vec{t}-{\vec{d}}_{c_{k}\left(i\right)}∥}_{2}^{2}+\alpha {\left(r_{i}^{m}-r_{c_{k}\left(i\right)}^{d}\right)}^{2}\\ +\alpha {\left(g_{i}^{m}-g_{c_{k}\left(i\right)}^{d}\right)}^{2}+\alpha {\left(b_{i}^{m}-b_{c_{k}\left(i\right)}^{d}\right)}^{2})/2\sigma^{2}) \end{array}$$

Assuming

(8) $$\begin{array}{c} {\vec{l}}_{i}\overset{\Delta }{\;=}\exp(-({∥A{\vec{m}}_{i}+\vec{t}-{\vec{d}}_{c_{k}\left(i\right)}∥}^{2}\\ +\alpha {\left(r_{i}^{m}-r_{c_{k}\left(i\right)}^{d}\right)}^{2}+\alpha {\left(g_{i}^{m}-g_{c_{k}\left(i\right)}^{d}\right)}^{2}+\alpha {\left(b_{i}^{m}-b_{c_{k}\left(i\right)}^{d}\right)}^{2})/2\sigma^{2}) \end{array}$$

By calculating the derivative of $\vec{t}$ for Equation (7), let $\frac{\partial F(A,\vec{t})}{\partial \vec{t}}=0$, we can get:

(9) $${\vec{t}}_{k}=\frac{\sum_{i=1}^{N_{m}}\left({\vec{d}}_{c_{k}\left(i\right)}-A_{k}{\vec{m}}_{i}\right)l_{i}}{\sum_{i=1}^{N_{m}}l_{i}}$$

The algorithm is solved iteratively, and the final loop termination is conditional on registration accuracy. Although the value of each iteration is an approximate solution, the final registration result will not be affected.

Second, we calculate the approximate solution of $A_{k}$. Substituting Equation (9) into (7), we can get a constraint optimization problem about $A_{k}$. Then, we can get:

(10) $$\begin{array}{c} F\left(A_{k}\right)=\underset{A}{\arg\max}\sum_{i=1}^{N_{m}}\exp(-({∥A{\vec{m}}_{i}+\frac{\sum_{i=1}^{N_{m}}\left({\vec{d}}_{c_{k}\left(i\right)}-A_{k}{\vec{m}}_{i}\right)l_{i}}{\sum_{i=1}^{N_{m}}l_{i}}-{\vec{d}}_{c_{k}\left(i\right)}∥}^{2}\\ +\sum_{i=1}^{N_{m}}(\alpha {\left(r_{i}^{m}-r_{c_{k}\left(i\right)}^{d}\right)}^{2}+\alpha {\left(g_{i}^{m}-g_{c_{k}\left(i\right)}^{d}\right)}^{2}+\alpha {\left(b_{i}^{m}-b_{c_{k}\left(i\right)}^{d}\right)}^{2}))/2\sigma^{2}) \end{array}$$

Let ${\vec{x}}_{i}={\vec{m}}_{i}-\frac{\sum_{i=1}^{N_{m}}{\vec{m}}_{i}l_{i}}{\sum_{i=1}^{N_{m}}l_{i}}$ and ${\vec{y}}_{i}={\vec{d}}_{c_{k}\left(i\right)}-\frac{\sum_{i=1}^{N_{m}}{\vec{d}}_{c_{k}\left(i\right)}l_{i}}{\sum_{i=1}^{N_{m}}l_{i}}$, we can get:

(11) $$\begin{array}{c} F\left(A_{k}\right)=\underset{A}{\arg\max}\sum_{i=1}^{N_{m}}\exp(-({∥A{\vec{x}}_{i}-{\vec{y}}_{i}∥}_{2}^{2}\\ +\sum_{i=1}^{N_{m}}(\alpha {\left(r_{i}^{m}-r_{c_{k}\left(i\right)}^{d}\right)}^{2}+\alpha {\left(g_{i}^{m}-g_{c_{k}\left(i\right)}^{d}\right)}^{2}+\alpha {\left(b_{i}^{m}-b_{c_{k}\left(i\right)}^{d}\right)}^{2}))/2\sigma^{2}) \end{array}$$

The process of solving $A_{k}$ is similar to[47,48], so we can solve $A_{k}$ in the same way, let *U* and *V* as $N_{m}\times \left(m+1\right)$ matrices where each row represents the point ${\vec{m}}_{i}$ and ${\vec{d}}_{c_{k}\left(i\right)}$, respectively, then we can get

(12) $$F\left(A_{k}\right)=\underset{A}{\arg\max}1_{N_{m}}^{T}G\left(V-A_{k}U\right)$$

where $1_{N_{d}}^{T}$ represents a row vector of length $N_{m}$, and $G\left(V-A_{k}U\right)=b$ represents a column vector with entries $G_{i}=\exp\left(-\left({∥A{\vec{x}}_{i}-{\vec{y}}_{i}∥}_{2}^{2}+\alpha {\left(r_{i}^{m}-r_{c_{k}\left(i\right)}^{d}\right)}^{2}+\alpha {\left(g_{i}^{m}-g_{c_{k}\left(i\right)}^{d}\right)}^{2}+\alpha {\left(b_{i}^{m}-b_{c_{k}\left(i\right)}^{d}\right)}^{2}\right)/2\sigma^{2}\right)$.

Then, we can get $A_{k}$ by calculation

(13) $$A_{k}=V^{T}D\left(b\right)U{\left(U^{T}D\left(b\right)U\right)}^{-1}$$

where D(b)=diag(b). The above algorithm is summarized in Table 1. Since the proposed algorithm is similar to the traditional affine ICP algorithm, the time complexity is also similar to the traditional affine ICP algorithm. The calculation time of the proposed algorithm is mainly in the search for the closest point of the first step. In this step, the search strategy of the k-d tree is used in this step, and step two hardly affects the calculation time of the algorithm. Therefore, the time complexity of the algorithm proposed in this paper is $O(N_{m}\ln N_{d})$. The code of our algorithm is implemented in MATLAB 2018. The PC environment is Intel (R) Core (TM) i5 3.00GHz, 4GB RAM.

## 4. Experimental Results

This section further verifies the accuracy and robustness of the proposed algorithm through experiments. First, the proposed algorithm is verified by simulation experiments, and we compare it with the ICP algorithm [15], the ICP algorithm with color information (CICP) [36], the scaling ICP algorithm (SICP) [49], the affine ICP algorithm (AICP) [50], and the affine algorithm with correntropy (ACICP) [51,52], GeoTransformer(Geo) [33], respectively. Among them, the simulation experiments include single objects and indoor scenes. In addition, we further test with real data.

### 4.1. Simulation Experiment

**Figure 2:**
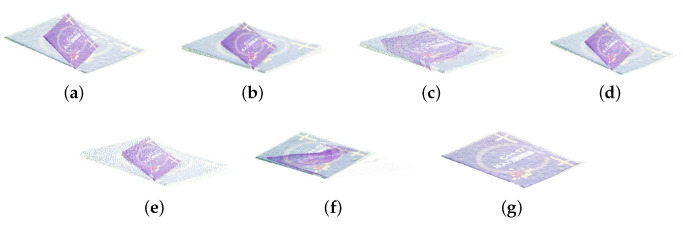
The registration results of the simulation experiment by different methods. (**a**) The original datasets. (**b**) ICP. (**c**) SICP. (**d**) CICP. (**e**) The affine ICP algorithm. (**f**) ACICP. (**g**) Ours.

**Figure 3:**
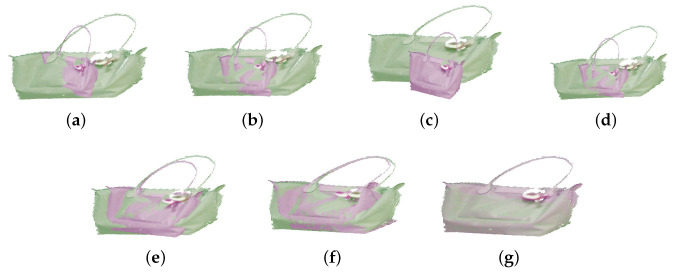
The registration results of the simulation experiment by different methods. (**a**) The original datasets. (**b**) ICP. (**c**) SICP. (**d**) CICP. (**e**) The affine ICP algorithm. (**f**) ACICP. (**g**) Ours.

**Figure 4:**
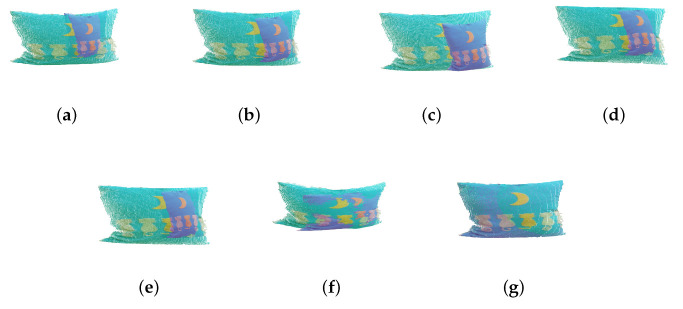
The registration results of the simulation experiment by different methods. (**a**) The original datasets. (**b**) ICP. (**c**) SICP. (**d**) CICP. (**e**) The affine ICP algorithm. (**f**) ACICP. (**g**) Ours.

In this section, we use simulation experiments to verify the accuracy and robustness of the algorithm in this paper. First, we use RGB-D data collected by Kinect [53,54,55], termed AffineMatch. Second, we add affine deformation and some noise to the point cloud data, and the data before and after the transformation are used for registration. Finally, when the ground-truth is known, the relative errors are computed. In the experiments, $\left\{A^{*},{\vec{t}}^{*}\right\}$ is the ground-truth, and the relative errors are defined as $\varepsilon_{A}=||A-A^{*}|{|}_{2}/\left|\right|A^{*}|{|}_{2}$, and $\varepsilon_{\vec{t}}=\left|\right|\vec{t}-{\vec{t}}^{*}|{|}_{2}/\left|\right|{\vec{t}}^{*}|{|}_{2}$. The experimental results are shown as Table 2 and Figure 2, Figure 3 and Figure 4.

**Figure 5:**
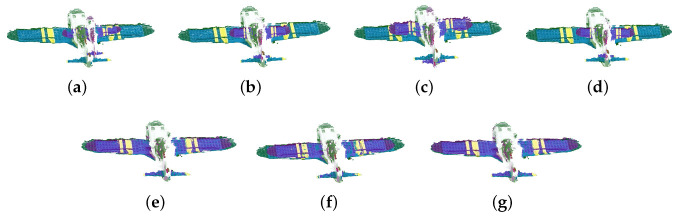
The registration results of the simulation experiment by different methods. (**a**) The original datasets. (**b**) ICP. (**c**) SICP. (**d**) CICP. (**e**) The affine ICP algorithm. (**f**) ACICP. (**g**) Ours.

As shown in Table 2, comparing the proposed algorithm with the other six algorithms, it is verified that the proposed algorithm is better than other algorithms. For the ACICP algorithm, although the correntropy criterion ensures the robustness of the algorithm to noise and outliers, it still cannot effectively align the two point sets in the absence of the correct correspondence between the point sets. Although the Geo [33] method is currently an excellent deep learning registration method, the registration accuracy for this affine deformation is low. The proposed algorithm performs well on simulated datasets with weak geometric structures with small errors. Figure 2, Figure 3, Figure 4 and Figure 5 are the result of simulation experiments on single objects, including books, bags, pillows, and airplanes. Obviously, the results of our algorithm are better than the registration results of the ICP algorithm, the ICP algorithm with correntropy, the affine algorithm with color information in terms of registration accuracy.

### 4.2. Indoor Scenes Experiment

**Figure 6:**
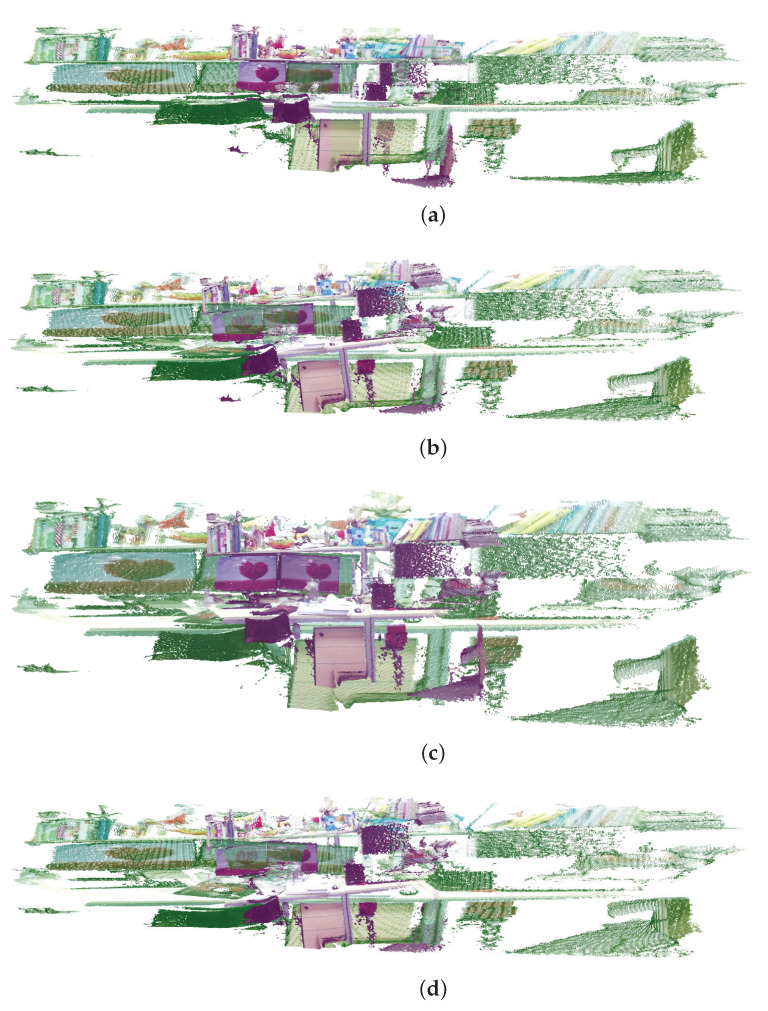

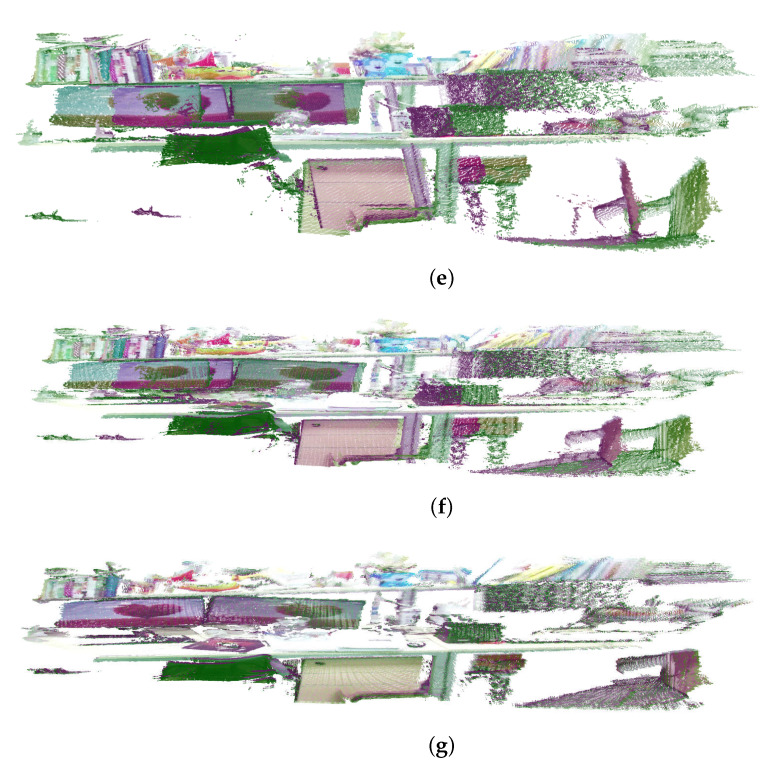
The registration results of the indoor scenes experiment by different methods. (**a**) The original datasets. (**b**) ICP. (**c**) SICP. (**d**) CICP. (**e**) The affine ICP algorithm. (**f**) ACICP. (**g**) Ours.

**Figure 7:**
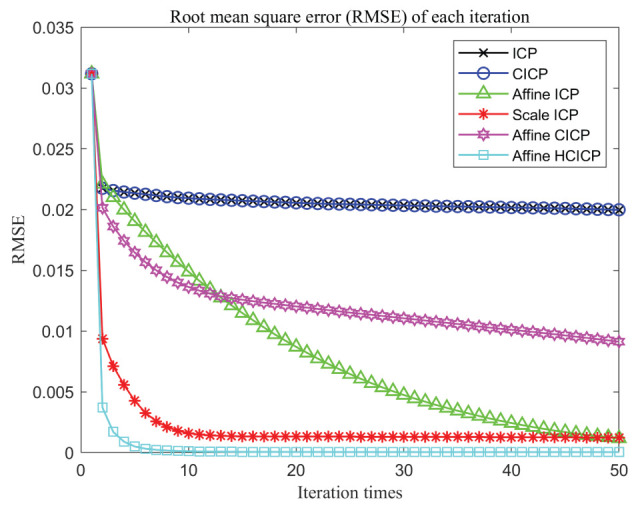
Comparison the RMS convergence results of indoor scene data with different algorithms.

In this section, we use indoor scenes experiments to verify the accuracy and robustness of the algorithm in this paper. The data are captured by a Microsoft Kinect sensor, which can simultaneously obtain RGB and depth images. We capture the real data in indoor scenarios and four point cloud pairs are used to perform registration experiments. The experimental results are shown as Table 3. As shown in Table 3, comparing the proposed algorithm with the other six algorithms, it is verified that the proposed algorithm is better than other algorithms. Figure 6 is the results with indoor data, where the red ones represent the source point set and the green ones represent the target point set. It can be seen from experiments that our algorithm is better than the other algorithms and can match more points, so it achieves a more accurate registration. Moreover, to further verify that the algorithm proposed in this paper may converge faster and obtain more accurate and robust registration results, we use indoor data to compare the convergence of the algorithm proposed in this paper with the other five algorithms, as shown in Figure 7. We define our algorithm as Affine HCICP. It can be seen from the figure that our algorithm has a faster convergence speed and stronger accuracy and robustness, which further proves that the proposed algorithm performs better.

### 4.3. Experiments with Real Data

In this section, we test on real data and further prove that the proposed algorithm is better than the other five algorithms. Since the real data do not have accurate ground-truth, and the real data contain noise and outliers, we use the root mean square error (RMS) method to analyze the accuracy of the proposed algorithm and other algorithms under the real data. First of all, because after the registration algorithm is matched, most of the points can find their corresponding points. After a lot of experiments, we select the first 60% of the points after registration to measure the registration accuracy of the algorithm. Secondly, we use the corresponding point pair as the root mean square error ε to measure the registration accuracy of the algorithm. As shown in Table 4, we compare different registration algorithms on two sets of real data. It can be obtained that our algorithm has higher matching accuracy and robustness compared to the other five algorithms.

**Figure 8:**
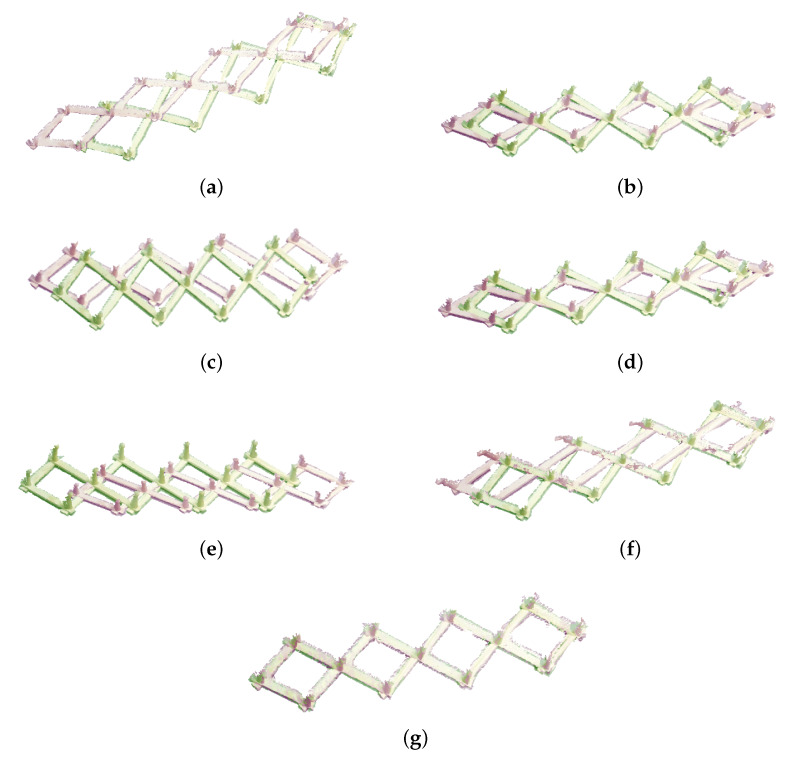
The registration results of the real data by different methods. (**a**) The original datasets. (**b**) ICP. (**c**) SICP. (**d**) CICP. (**e**) The affine ICP algorithm. (**f**) ACICP. (**g**) Ours.

**Figure 9:**
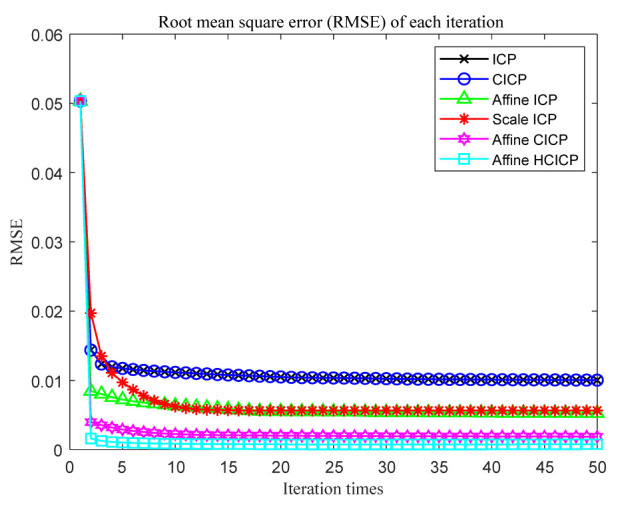
Comparison the RMS convergence results of real data with different algorithms.

In addition, as shown in Figure 8, when tested on a real dataset, we can see that the proposed algorithm can match two point sets well, while the registration accuracy of other algorithms is not as good as the proposed algorithm. Thus, the accuracy and robustness of the proposed algorithm are verified. Moreover, to further verify that the proposed algorithm converges faster on real datasets and obtains more accurate and robust registration results, we use real data to conduct the convergence of the proposed algorithm with the other five algorithms. As shown in Figure 9, it can be seen from the test on real data that our algorithm has faster convergence speed, stronger accuracy, and robustness, which further proves that the algorithm performs better.

## 5. Conclusions

This paper proposes a precise affine registration algorithm with correntropy and color information. This algorithm can effectively handle the large noise and outliers and small deformation in the dataset in the registration algorithm. First, the algorithm introduces color features to establish more accurate and reliable correspondence. Second, this paper introduces the measurement of correntropy, which overcomes the influence of noise and outliers in the dataset, and further improves the registration accuracy. Experimental results demonstrate that our registration algorithm has higher registration accuracy and more stable robustness than other advanced algorithms. In the future, the robustness against illumination changes of the algorithm can be further studied.

## Figures and Tables

**Table 1:** The precise affine registration algorithm with color information and correntropy.

Thepreciseaffineregistrationalgorithmwithcolorinformationand
correntropy
Input:TwopointsetsM and D
Initialization:Theinitialaffinetransformation$A_{0},{\vec{t}}_{0}$.
Repeattwosteps:
Step1:Accordingtothetransformationinstep(k−1),thecorresponding
relationshipisestablishedbetweenthetwopointsetsviaEquation(5).
Step2:Calculating$\left(A_{k},{\vec{t}}_{k}\right)$ via Equation (6).
End
Output: $A_{k},{\vec{t}}_{k}$

**Table 2:** Comparison of errors with simulation data by different methods.

Data	Error	ICP	SICP	CICP	AICP	ACICP	Geo	Ours
1	$\varepsilon_{A}$	4.2404	2.4567	4.2396	0.8909	0.4171	4.1203	0.0027
	$\varepsilon_{\vec{t}}$	0.0047	0.0063	0.0046	0.0076	0.0024	0.0049	0.0001
2	$\varepsilon_{A}$	2.3110	2.7573	2.3110	9.1136	185.5356	2.2430	0.0017
	$\varepsilon_{\vec{t}}$	0.0046	0.2191	0.0046	3.7153	73.5416	0.0045	0.0017
3	$\varepsilon_{A}$	2.4192	2.3792	2.4047	0.5648	1.3316	2.3182	0.0002
	$\varepsilon_{\vec{t}}$	0.0405	0.1401	0.0338	0.0533	0.7607	0.0389	9.9 × $10^{-6}$
4	$\varepsilon_{A}$	2.3729	2.3284	2.3424	2.7484	1.4589	2.2894	0.0002
	$\varepsilon_{\vec{t}}$	0.0187	0.1071	0.0070	2.8215	1.1970	0.0172	0.0001

**Table 3:** Comparison of errors with indoor datasets by different methods.

Data	Error	ICP	SICP	CICP	AICP	ACICP	Geo	Ours
1	$\varepsilon_{A}$	2.4466	2.0499	2.4452	0.0188	0.4177	2.4424	0.0131
	$\varepsilon_{\vec{t}}$	0.0055	0.0048	0.0005	0.0076	0.0002	0.0047	0.0003
2	$\varepsilon_{A}$	4.2451	1.5872	4.1448	0.0257	9.2455	4.3012	0.0248
	$\varepsilon_{\vec{t}}$	0.0089	0.0252	0.0086	0.0006	0.2515	0.0078	0.0004
3	$\varepsilon_{A}$	2.4497	1.8683	2.4526	0.0767	0.0728	2.3342	0.0542
	$\varepsilon_{\vec{t}}$	0.0010	0.0021	0.0012	0.0003	0.0002	0.0009	0.0002
4	$\varepsilon_{A}$	4.0085	1.8849	4.0209	0.0893	0.0979	4.0143	0.0570
	$\varepsilon_{\vec{t}}$	0.0027	0.0245	0.0032	0.0007	0.0010	0.0031	0.0007

**Table 4:** Comparison of errors by different methods.

	The Error ε of Data 1	The Error ε of Data 2
ICP	1.7 × $10^{-5}$	2.0 × $10^{-4}$
SICP	1.7 × $10^{-5}$	2.0 × $10^{-4}$
CICP	8.9 × $10^{-5}$	3.4 × $10^{-4}$
AICP	3.1 × $10^{-3}$	1.5 × $10^{-3}$
ACICP	8.8 × $10^{-5}$	1.1 × $10^{-4}$
Ours	8.2 × $10^{-6}$	6.7 × $10^{-6}$

## Data Availability

Trained models with algorithm can be available upon reasonablerequest according to the instructions in ICP algorithm [15], the ICP algorithm with color information (CICP) [36], the scaling ICP algorithm (SICP) [49], the affine ICP algorithm (AICP) [50], and the affine algorithm with correntropy (ACICP) [51,52], GeoTransformer(Geo) [33].
